# High density lipoproteins-based therapies for cardiovascular disease

**DOI:** 10.4103/0975-3583.70898

**Published:** 2010

**Authors:** Xuan Gao, Shujun Yuan

**Affiliations:** *Departments of Physiology and Biophysics, Boston University School of Medicine, 700 Albany St. W302, Boston, MA 02118, USA*

**Keywords:** High density lipoprotein, reverse cholesterol transport, atherosclerosis, drug development

## Abstract

Atherosclerosis is the leading cause of death in developed countries. High density lipoproteins (HDL) cholesterol level correlates inversely with the risk of cardiovascular diseases. Thus, HDL has obtained lots of interest for drug development. In this review, we summarized the mechanisms for the antiatherogenic function of HDL, current HDL-based drugs in clinical use and the future direction for HDL-based therapy development.

## INTRODUCTION

Cardiovascular disease (CVD) is the number one killer disease in the United States and most other developed countries.[1] Plasma level of cholesterol is the key indicator for the risk of developing CVD. Low-density lipoprotein cholesterol (LDL-C) and high-density lipoprotein cholesterol (HDL-C) levels are two independent risk factors.[2,3] High concentration of LDL-C is strongly associated with cardiovascular disease. In contrast, HDL-C correlates inversely with the risk of CVD.[2,3] The National Cholesterol Education Program (NCEP) Adult Treatment Panel III guidelines have recognized low HDL-C (<40 mg/dL) as an independent major risk factor for CVD.[4] Currently, statins are the most widely used drugs in modifying the cholesterol level. Statins inhibit 3-hydroxy-3-methyglutaryl-coenzyme A (HMG-CoA) reductase activity, and, thus, inhibits cholesterol synthesis in the liver.[5] As a result, expression of LDL receptors is boosted to facilitate the removal of LDL from circulation.[5] Therefore, it is believed that the anti-atherosclerotic function of statins is mainly due to their abilities to lower plasma LDL-C.[6,7] However, despite the wide use of statins, CVD remains the leading cause of death in industrialized countries. Therefore, other lipid-modifying therapeutic strategies are being sought to improve the treatment of atherosclerosis. This includes an increasing interest in using HDL as a therapeutic target.

## HDL COMPOSITION, STRUCTURE, AND FUNCTION

HDL are heterogeneous complexes of proteins and lipids differing in shape (nascent discoidal or mature spherical), diameter (8–13 nm), density (1.21–1.063 g/ml), protein and lipid composition, and function.[8] Nascent discoidal HDL are comprised of a cholesterol-containing phospholipid bilayer surrounded by apolipoprotein α-helices.[9] Mature spherical HDL are composed of a monolayer of phospholipids and helical proteins on the surface and cholesterol ester as well as a small amount of triglycerides in the hydrophobic core.[10] Apolipoprotein A-I (apoA-I) is the major protein on HDL that accounts for ~70% of total HDL proteins. The second major protein, apoA-II, represents ~20%, and other HDL proteins, including apoE, apoA-IV, apoA-V, apoJ, apoC-I, apoC-II, and apoC-III, account for <10% of the HDL protein content.[11] Based on their density, mature HDL can be further divided into HDL_2_ (1.063–1.125 g/ml) and HDL_3_ (1.125–1.21 g/ml). These subclasses vary in protein and lipid composition and have different functions. For example, smaller HDL_3_ are relatively enriched in apoA-II.[12] Importantly, HDL_2_ and HDL _3_ have distinct functional and metabolic properties. Large HDL_2_ are believed to be more atheroprotective than small HDL_3_, because epidemiologic studies show that, compared with healthy individuals, patients with high risks of CVD often have higher levels of small HDL_3_ but lower level of large HDL_2_.[13] Also, HDL_2_ have been reported to have an enhanced ability to mediate cholesterol ester uptake via the scavenger receptor type B1 (SR-B1),[14] which is expected to contribute to their cardioprotective action.

HDL protect against atherosclerosis mainly by playing a central role in reverse cholesterol transport (RCT). RCT involves removal of cholesterol from arterial macrophages and delivery of this excess cholesterol to the liver for excretion or to steroidogenic organs for hormone synthesis, thereby preventing the formation of arterial plaques.[15] Since RCT is the only cholesterol-removal pathway in human, the role of HDL in cadioprotection is indispensable. Cholesterol efflux mediated by lipid-free or lipid-poor apoA-I through ABCA1 is the most efficient cholesterol removal pathway.[16] In the presence of lecithin:cholesterol acyltransferase (LCAT), cholesterol in HDL surface is esterified. The apolar molecules of cholesterol ester move from the surface to the core of the particle. As a result, mature HDL acquire their spherical shapes. Small spherical HDL_3_ can take up additional cholesterol and are further remodeled by LCAT and other plasma factors. Subsequently, they fuse into larger HDL_2_. HDL finally deliver their cargo of cholesterol to the liver for excretion through scavenger receptor class B type I (SR-BI). Although lipid-free or lipid-poor apoA-I is the primary cell cholesterol acceptor, spherical HDL also pick up cholesterol from peripheral tissues. Several ABC transporters, including ABCG1 and ABCG4, are involved in cholesterol efflux from macrophages to HDL_2_ and HDL_3_.[17] Moreover, SR-BI is proposed to mediate bidirectional flux of cholesterol between mature HDL and cells. Thus, SR-BI can promote cholesterol efflux into HDL when a concentration gradient exists.[11]

In addition to their central role in RCT, HDL also have antioxidant, anti-inflammatory and anti-thrombotic properties, which contributes to their anti-atherogenic action. The anti-oxidant property of HDL is mainly due to their ability to inhibit LDL oxidation and remove oxidized lipids from LDL. According to the oxidation hypothesis of atherogenesis, oxidized LDL is more readily taken up by macrophages, which promotes foam cell formation. Therefore, deoxidization of LDL can decrease the risk of arterial plaque formation.

The anti-inflammatory property of HDL is due to their ability to inhibit the expression of adhesion molecules, such as vascular cell adhesion molecule 1, intercellular adhesion molecule 1, and E-selection,[18] thereby inhibiting monocyte adhesion to endothelial cells, infiltration into the arterial wall, and maturation to macrophage. The mechanism of inhibition may involve apoA-I, apoA-II, apoA-IV, and phospholipids such as sphigosine-1-phosphate and sphingosylphosphorylcholine.[18] As oxidized lipids are pro-inflammatory,[19] the ability of HDL to remove these lipids also contributes to their anti-inflammatory action.

The anti-thrombotic action of HDL is due to the inhibition of factors that promote blood coagulation, including factors X, Va, and VIIIa. This inhibition may result from anionic HDL lipids cardiolipin and phosphatidylethanolamine, which have anticoagulant properties.[11]

## HDL-BASED THERAPIES

Statins have been reported to moderately increase the HDL-C level by 3–13%.[20] This HDL-C increasing effect is significantly smaller than the statin-induced LDL-C lowering effect. Thus, the benefits of statin-induced HDL-C elevation are unclear and statins normally are not considered as HDL-raising drugs. Currently, niacin and fibrates are the main HDL-targeting therapies.

### Niacin

To date, niacin (Nicotinic acid, Vitamin B3) is the most effective drug in the market to increase HDL-C (by 15–30%).[21] Although niacin is available from many dietary sources, only high dose of niacin (1.5–2 g/day) has HDL-C elevation effects.[22] Many clinical trials have demonstrated that the niacin-induced increase in HDL-C level is associated with reduced risk of CVD. These clinical trails include Coronary Drug Project (CDP),[23] Familial Atherosclerosis Treatment Study (FATS),[24] HDL Atherosclerosis Treatment Study (HATS),[25] and, more recently, the randomized Arterial Biology for the Investigation of the Treatment Effects of Reducing Cholesterol Trials (ARBITER-2 and ARBITER-3).[26,27]

The beneficial effect of niacin in modifying cholesterol levels was discovered 50 years ago,[28] but the underlying mechanism began to be elucidated only recently. Niacin activates the niacin receptor HM74A (GPR109B) in adipocytes, which inhibits adenylyl cyclase. Consequently, the amount of free fatty acid released from adipocytes to circulation is decreased, which leads to diminished plasma and hepatic triglyceride levels. As a result, HDL-C is increased.[29] However, the side effect of flushing compromised the wide use of niacin. Extended-release (ER) formulations of niacin, which cause less flushing because of slower release of niacin, are available, but some of them seem to have increased liver toxicity.[30] Niacin causes flushing by elevating the synthesis of prostaglandin D2, which activates DP1 receptor and thus acts as a vasodilator.[31] Laropiprant, a DP1 antagonist, was developed by Merck & Co. for use in combination with niacin to minimize flushing. Laropiprant was approved by the European Medicines Agency, but not by the FDA, since administration of 325 mg aspirin, a widely used non-steroidal antiinflammatory drug, 30–60 min before niacin can also reduce flushing.[32]

### Fibrates

Fibrates are the other HDL-C raising drugs (by 5–20%) in the market. Fibrates indirectly raise plasma HDL level and lower triglyceride concentration through activating nuclear transcription factor PPARα. It has been proposed that fibrates increase HDL level through upregulation of apoA-I and ABCA1 and downregulation of SR-B1. Many clinical trials showed that fibrates are beneficial for both primary and secondary prevention of CVDs, including the Helsinki Heart Study (HHS) (600 mg of gemfibrozil twice daily) and Veterans Affairs HDL Intervention Trial (VA-HIT) (1.2 g/day gemfibrozil).[33,34] However, effects of fibrates on CVD seem to be uncertain; for example, the Fenofibrate Intervention and Event Lowering in Diabetes (FIELD) trial (fenofibrate 200 mg/day) failed to demonstrate beneficial effects of fibrates.[35]

## FUTURE DIRECTIONS FOR HDL-BASED DRUGS

### ApoA-I mimetic peptides

ApoA-I mimetic peptides are amphipathic peptides of 18–22 amino acids, which mimic the lipid-binding domain of apoA-I. ApoA-I mimetics retain functional properties of apoA-I, such as the ability to form complexes with lipids, promote cell cholesterol efflux, and activate LCAT.[36] The advantage of mimetic peptides over the full-length apoA-I is that they are relatively easy and cheap to synthesize. D4F, an orally active peptide with D-amino acids (to avoid proteolysis by gut peptidases that recognize L-amino acids) and 4 phenylalanine substitutions, has been shown to enhance the anti-oxidant and anti-inflammatory function of HDL and improve its cholesterol efflux ability without increasing plasma HDL-C levels in mouse models.[36] However, D4F seems to show toxicity in human trials.

### CETP inhibitor

Cholesterol ester transfer protein (CETP) mediates equimolar exchange of cholesterol ester for triglyceride among HDL and the triglyceride-rich apoB-containing lipoproteins (VLDL, IDL, LDL, chylomicrons, and their remnants).[37] The net effect of CETP action on HDL is enrichment with triglyceride and depletion of cholesterol ester.[37] As CETP reduces the HDL-C level, therapeutic approaches targeting CETP inhibition have been of interest for years.[37] Indeed, humans with CETP deficiency have higher levels of HDL-C and apoA-I and tend to have lower rates of atherosclerosis.[38] However, the clinical trials of torcetrapib, a CETP inhibitor developed by Pfizer, were halted in 2006 due to an interim finding of Investigation of Lipid Level Management to Understand Its Impact in Atherosclerotic Events (ILLUMINATE) trail.[39] Even though torcetrapib elevated the HDL-C level, it unexpectedly increased the risk for atherosclerotic events and led to a higher incidence of cardiovascular and non-cardiovascular death.[39] It is not entirely clear whether the increased cardiovascular risk was due to the CETP inhibition or to the off-target effects of torcetrapib.[40] Currently, two other CETP inhibitors, Dalcetrapib (JTT-705) and Anacetrapib (MK-0859), are still in clinical trials. In contrast to torcetrapib, Dalcetrapib and Anacetrapib increase HDL-C without increasing blood pressure, but further clinical trials are required to evaluate their efficacy and safety.[41,42]

The failure of torcetrapib suggests that raising HDL-C levels alone may not necessarily provide protection from atherosclerosis.[13] Growing clinical evidence suggests that both HDL quantity and quality are important for cardioprotection. Epidemiological studies show that larger HDL are more cardioprotective than smaller HDL.[13] Functional studies revealed that mild oxidation benefits HDL function in cholesterol efflux while extensive oxidation impairs this function.[43] Studies by Gursky and colleagues suggest that less stable HDL has better functional properties and is more cardioprotective.[44–46] Therefore, measuring HDL stability may provide a simple way to assess HDL quality.

### Lipase inhibitors

Hepatic lipase (HL)[7] and endothelial lipase (EL) can hydrolyze HDL triglycerides and phospholipids, leading to generation of lipid-poor apoA-I, which is susceptible to degradation. Thus, inhibition of HL and EL was proposed to increase the plasma HDL level.[13,47] However, HL can also clear atherogenic apoB-containing particles; therefore, therapeutic strategies involving HL inhibition should be treated with caution. On the other hand, elevated EL levels are reportedly associated with atherosclerosis in humans.[47] More research is needed to determine the potential of using EL inhibitors to raise the plasma level of HDL.

### Liver X receptor agonists

Liver X receptor(LXR), one of the ligand-activated transcription factors, belongs to the nuclear receptor family. LXR regulates genes in cholesterol efflux (ABCA1, ABCG1, ABCG4), genes in HDL remodeling (CETP and phospholipid transfer protein), genes in cholesterol secretion and bile-acid synthesis, and genes in hepatic lipogenesis.[30] Animal studies have shown that LXR activation leads to elevated HDL-C, and also elevated triglycerides, which may lead to the development of fatty liver.[13,48] Thus, the ideal LXR agonist would selectively upregulate ABCA1 and ABCG transporters in macrophages to promote reverse cholesterol transport and selectively upregulate genes in cholesterol secretion without activating genes in hepatic lipogenesis. LXRs have two isoforms, LXRα and LXRβ. LXRα is more abundant in the liver while LXRβ is ubiquitously expressed. Thus, selective modulation of LXRβ but not LXRα may lead to elevated HDL-C levels without the risk of developing hypertriglyceridemia and fatty liver.

## CONCLUSIONS

LDL-C reducing drugs have been clinically used for decades, but the incidence of CVD is still high. Thus, increasing HDL level to complement statins, the LDL-lowering drugs, became an attractive target. Despite vigorous efforts, HDL-modifying drugs are still not effective in decreasing mortality. In addition, the failure of CETP inhibitor torcetrapib casts a shadow on HDL-based drug development. Most HDL-based drug development efforts were focused on HDL-C increase. Recent studies showed that both the quantity and the quality of HDL are important for cardioprotection. It is possible that improving various aspects of HDL function will become a new direction for the development of HDL-based therapies in the future. Thus, not only increasing HDL-C level but also increasing functionally improved HDL may be a more effective strategy for CVD treatment.
